# Decision‐Making and Family Planning among People with Clinical Depressiveness—Findings from a Representative German Panel Study

**DOI:** 10.1111/sifp.70060

**Published:** 2026-07-15

**Authors:** Marie Lehnert, Tanja Gabriele Baudson, Doris Erbe

## Abstract

Personally important decisions, like reproductive choices, may be challenging for individuals with clinical depressiveness, as indecisiveness is a common depressive symptom. This study examines whether decision‐making modes related to reproductive behavior differ between individuals with and without clinical depressiveness (H1) and whether these modes remain stable over time, even after the birth of the first child (H2). Data stem from the German Panel Analysis of Intimate Relationships and Family Dynamics (*pairfam*). Clinical depressiveness was measured using State‐Trait Depression Scales and decision‐making modes through items on reproductive behavior. H1 was analyzed using multivariate analysis of covariance (MANCOVA) and post hoc analysis of variance (ANOVA); H2 via fixed‐effect regressions and cross‐lagged panel model. A significant group difference emerged in the combined decision‐making modes (Pillai's trace = 0.009, *F*(6, 4832) = 7.32, *p* < 0.001). Individuals with clinical depressiveness engaged more in rational and avoidant modes, whereas those without favored normative decision‐making. Decision‐making remained stable between subjects; within‐subject analyses showed alterations over time. Between subjects, decision‐making remained stable. Characteristics of depressive symptoms and individual circumstances may explain the results. Future research should incorporate other clinical conditions and qualitative analyses. Practitioners should consider individual costs, benefits, and decisional postponement.

## INTRODUCTION

Despite increasing mental health awareness in recent years (e.g., Foulkes and Andrews 2023), family planning remains a taboo topic for individuals with mental disorders (Krumm and Becker 2006; Schonewille 2024). They may face challenges across several domains, including reproductive history, reproductive decision‐making process, parenting style, and sexuality (Schonewille et al. 2023). For instance, unplanned pregnancies are more common in mentally vulnerable people (Schonewille et al. 2022), and there are higher inconsistencies in contraception usage (Kremer, Gerlach, and Erbe 2024). In addition, parents with depressive symptoms are more likely to regret their decision to become parents (Erbe, Freisinger, and Gerlach 2025). Still, there is a gap in the perceived relevance and the proactive inquiry of reproductive topics by professionals during therapy (Engel et al. 2026).

In case individuals with mental illnesses do receive counseling on family planning, the provided guidance is often directive and lacking a participatory decision‐making process (Schonewille 2024). Oftentimes, healthcare professionals focus primarily on contraceptive education (Krumm and Becker 2006) and perceive personal life circumstances as aspects not subject to shared decision‐making (Scherer and Alpers 2025). They may even perceive mental illness as a risk factor, and rather advise against having children than engage in a sensitive, empathic discussion (Krumm and Becker 2006). Inevitably, there is a shortage of individualized and sensitive family planning guidance for men and women with mental health issues (e.g., Schonewille 2024; Schonewille et al. 2022). Even nonclinical samples report unmet counseling needs regarding decisions about parenthood (Meyer, Ehrenthal, and Erbe 2024). In contrast, well‐planned family arrangements contain protective factors for mentally ill parents like normalization of their life situation, self‐development, purpose, and feeling loved (Krumm and Becker 2006). Thus, psychosocial support concerning parenting decisions of individuals with mental disorders is an important topic for preventing further distress in parents.

Depression, as a mental disorder, manifests as changes in the patient's thoughts, feelings, and behavior (Bebbington 2001). These changes are recognizable in symptoms such as negative thoughts, listlessness, a pessimistic perspective of the future, feelings of sadness, guilt and indifference, impaired cognitive functions, or isolation, as well as insecurity and indecisiveness (Freyberger, Dilling, and Weltgesundheitsorganisation 2019). Because decision‐making processes are shaped by one's beliefs (Huys and Dayan 2009), skills, and emotions (Paulus and Yu 2012), depressive symptoms may influence these processes. Often, people with depression experience decisional conflict—an uncertainty regarding decisions. It manifests in feeling uninformed, worried, stressed, questioning one's values, as well as putting off or ruminating about the decision (Stacey et al. 2008). But, initiating the decision‐making process requires certainty regarding the decisional outcome (Leahy 2001). While people with depression often wish to take an active role in the decision‐making process, uncertainty is associated with less active involvement in decisions (Stacey et al. 2008). Decisions are often less productive and less in line with the decision‐maker's interests (Leykin, Roberts, and DeRubeis 2011), which may be related to avoiding further decisions. Feelings of helplessness are associated with difficulties in making contrasting experiences (Abramson, Seligman, and Teasdale 1978), which might lead to doubts after the decision is made.

As individuals with depressive symptoms repeatedly experience difficulties making decisions in general (Hindmarch, Hotopf, and Owen 2013), it can be assumed that this also applies to decisions regarding family planning. Parenthood decisions incorporate if, when, with whom, and under which circumstances people decide to become parents, as well as their preferred number of children (Meyer, Ehrenthal, and Erbe 2024). Thus, they illustrate a complex and individual decision‐making process (in the present study referred to as decision‐making mode) addressing various important factors and life circumstances. Parenthood decisions integrate, among others, values that are attributed to having children. If having a family is based on one's core values, the decisions should not be affected by depressive symptoms, as values are expected to be generally stable (Vecchione et al. 2016). Though values evaluated during the individual decision‐making process may alter in relation to emotional belief systems (Paulus and Yu 2012). Simultaneously, life changes such as the birth of a child have been linked to subsequent reproductive decisions (Cassé et al. 2018). Decisions embedded in a value‐based belief system may be stable over time, whereas situation‐based emotions and developed skills may be related to those decisions.

In conclusion, people with depression face challenges while making decisions due to cognitive, behavioral, and emotional changes associated with depressive symptoms. Decisional conflicts and dysfunctional decision‐making styles may also apply in the context of reproductive decisions. Thus, depression‐related characteristics may be associated with differences in the decision‐making process in a way that does not necessarily apply to individuals without depressive symptoms. The decisions made during the family planning process may be stable over time since they are made on a strong value‐based belief system.

## HYPOTHESES

Summarizing findings of current research, we propose the following research question and hypotheses:

Do decision‐making modes related to reproductive behavior differ between individuals with and without clinical depressiveness and do they remain stable over time?

H 1 Decision‐making modes related to reproductive behavior differ between individuals with and without clinical depressiveness.

H 2 : Decision‐making modes remain stable over time for individuals with and without clinical depressiveness, both prior to and following the first birth.

## METHODS

The present study examines the attitudes of individuals with and without clinical depressiveness regarding decision‐making processes and their stability. It analyzes data from the German Panel Analysis of Intimate Relationships and Family Dynamics (*pairfam*; Brüderl et al. 2024; Huinink et al. 2011). All analyses were conducted under the terms and privacy regulations of *pairfam*. *Pairfam* was approved by the ethics committee of the Faculty of Management, Economics and Social Sciences of the University of Cologne, and all participants consented to the participation. All procedures contributing to this work comply with the ethical standards of the relevant national and institutional committees on human experimentation, and with the Declaration of Helsinki of 1964, as revised in 2024. *Pairfam* is an interdisciplinary panel survey that started in 2008 with over 12,402 participants. It collects yearly in‐person interview data on categories such as household, relationships, family, health, sexuality, parenthood, and personality. Since 2022, the *pairfam* study has been continued as the *Freda* study. For the proposed hypotheses, data on clinical depressiveness, decision‐making modes, as well as several covariates (age, sex, highest education, marital status, time of birth of first child) were analyzed.

Since the *pairfam* study contains a big sample that fluctuates across survey waves, a partial sample was drawn for the present study. We analyzed participants from Waves 2, 6, and 10, since the decision‐making modes were assessed exclusively during these periods. For the analysis of both hypotheses, participants from Wave 2 who did not have any children up to that point and who had no missing data in the variables were included. Wave 2 entails the first examination of the decision‐making modes. Thus, analyzing data from this time point did not include any recollection errors by the participants. We focused on childless participants from Wave 2 to ensure objectivity and comparability and to exclusively examine the effect of the first child. The overall sample size and the sample size of childless participants were the largest in Wave 2, compared to Waves 6 and 10. A sample of 4,839 participants was examined for the analyses of Hypothesis 1, with 278 participants with and 4,561 participants without clinical depressiveness. The analyses of Hypothesis 2 included participants from Waves 2, 6, and 10. Since only participants with no missing data in all three survey waves were included, the sample contained 1,552 participants (see Table 1).

**TABLE 1:** Descriptive statistics of the drawn samples

	Hypothesis 1		Hypothesis 2		
		Wave 2	Wave 2	Wave 6	Wave 10
*N*		4,839		1,552	
					
Cohort					
	1	2,905		917	
	2	1,478		537	
	3	456		98	
Sex					
	Female	2,516		805	
	Male	2,323		747	
CD					
	Yes	278	79	149	159
	No	4,561	1,473	1,403	1,393
STDS‐t					
	*M (SD)*	16.36 (4.41)	17.24 (4.73)		
Age					
	*M (SD)*	22 (6.7)	25.72 (6.98)		
Married					
	Yes	276	83	242	409
	No	4,563	1,465	1,306	1,138
	NA	‐	4	4	5
Children					
	Yes	0	0	200	412
	No	4,839	1,552	1,352	1,140

NOTE: CD = Clinical Depressiveness, M = Mean, SD = standard deviation.

Depressiveness was measured via the German version of the *State‐Trait‐Depression‐Scales* (STDS‐t) by Spaderna, Schmukle, and Krohne (2002). It is a screening tool and assesses the severity of depressive symptoms. The questionnaire consists of 10 statements, such as “*I'm sad.”*, *“My mood is melancholy.”*, *“I'm happy.”* with five inverted items (2, 7, 8, 9, 10). All statements are rated via a 4‐point Likert scale (“almost never” to “almost always”). A cutoff ≥ 25 indicates clinically relevant depression (Lehr et al. 2008). However, since no clinical assessment was conducted in the present study, we used the term *clinical depressiveness*. We created a dichotomous variable to differentiate between clinical and nonclinical depressiveness. Hence, two groups were created, one with individuals with clinical depressive symptoms and one with individuals without clinical depressive symptoms. In *pairfam*, all participants received the STDS‐t in Waves 2–14, with Wave 14 including only three items.

The decision‐making modes regarding parenthood describe the process by which the decision is made, regardless of whether the decision is for or against children. These modes were measured using the following question: “Below are some statements about how people make a decision for or against becoming a parent. To what extent do you agree personally with these statements?”. Six statements representing various modes (see Table 2) are rated on a 5‐point Likert scale (“disagree completely” to “agree completely”). The different decision‐making modes were developed during the *pairfam* minipanel (Brüderl et al. 2007). In the *pairfam* study, the decision‐making modes were assessed in Waves 2, 6, and 10. All participants who did not report infertility and who answered the question about their realistically expected number of children received this question. Starting from Wave 10, the question was no longer asked to women in the oldest cohort who do not plan to have children. For pragmatic reasons, the decision‐making modes were mainly categorized according to the general decision‐making styles model (GDMS) by Scott and Bruce (1995) in the present study.

**TABLE 2:** Decision‐making modes in pairfam grouped in categories based on the GDMS by Scott and Bruce (1995)

	Decision‐making mode in Pairfam	Category on the basis of the GDMS
1	Having children is a normal part of life	Normative mode
2	Personal costs‐and‐benefits driven decision	Rational mode
3	Emotionally guided	Intuitive mode
4	Partner should decide	Dependent mode
5	I put off the decision	Avoidant mode
6	Is not something you can plan	Spontaneous mode

In the present study, quantitative and longitudinal data were analyzed using *R* (version 4.4.0; R Core Team 2021). Generative AI (ChatGPT, OpenAI) was used to assist with the development and debugging of R scripts. However, data analysis, interpretation, and all substantive decisions were made entirely by the authors. Hypothesis 1 was analyzed via multivariate analysis of covariance (MANCOVA), including age, sex, highest education, and marital status as covariates. Hypothesis 2 was analyzed via fixed regression analyses, using the birth of the first child as a fixed effect. A time‐series analysis via a cross‐lagged panel model (CLPM; Campbell 1963) examined chronological predictions. To analyze the requirements of the statistical analyses, QQ plots and Henze–Zirkler test (Henze and Zirkler 1990) were used to examine multivariate and normal distribution of the dependent variables and the residuals. Bootstrapping (Efron and Tibshirani 1994) will be used against violations of normal distribution, even though, according to the central limit theorem, *n* > 30 is robust against such violations. To examine homoscedasticity, the Levene test, Breusch–Pagan test (Breusch and Pagan 1979), and Box‐M test (Box 1949) were used. The Durbin–Watson test (Durbin and Watson 1950) was used to measure autocorrelation and the Variance Inflation Factor (VIF)  for assessing multicollinearity.

## RESULTS

### Hypothesis 1

Descriptive analyses of each decision‐making mode show almost even distributions in relation to sociodemographic variables. The means of decision‐making modes 4–6 are generally lower than the means of modes 1–3. The distribution across the different sociodemographic variables is quite regular (see Table 3).

**TABLE 3:** Means (M) and standard deviations (SD) per decision‐making mode

	Mode 1	Mode 2	Mode 3	Mode 4	Mode 5	Mode 6
	*M* (*SD*)	*M* (*SD*)	*M* (*SD*)	*M* (*SD*)	*M* (*SD*)	*M* (*SD*)
Sex						
Male	3.87 (1.23)	2.93 (1.16)	3.34 (1.18)	1.97 (1.01)	1.67 (0.97)	2.60 (1.23)
Female	3.87 (1.31)	3.08 (1.20)	3.45 (1.22)	1.44 (0.74)	1.55 (0.96)	2.54 (1.27)
Cohort						
1	3.93 (1.20)	3.05 (1.11)	3.44 (1.16)	1.78 (0.93)	1.60 (0.93)	2.62 (1.22)
2	4.03 (1.20)	2.93 (1.25)	3.33 (1.24)	1.63 (0.90)	1.59 (0.98)	2.49 (1.24)
3	3.00 (1.53)	2.88 (1.37)	3.29 (1.32)	1.60 (0.96)	1.73 (1.14)	2.54 (1.42)
Married						
No	3.87 (1.26)	3.00 (1.17)	3.40 (1.19)	1.73 (0.93)	1.61 (0.96)	2.57 (1.24)
Yes	3.89 (1.43)	2.95 (1.37)	3.24 (1.30)	1.55 (0.85)	1.59 (1.05)	2.48 (1.35)

NOTE: *N* = 4,839.

Requirements for the MANCOVA were partially met (see the Online Appendix for all results). There was no multivariate normal distribution (*p* < 0.001); thus, bootstrapping was used. Most dependent variables showed no homoscedasticity, and no homoscedasticity was found in the variance–covariance matrix (Levene test: *p* < 0.001; BoxM test: *p* < 0.001). Thus, robust standard errors, Welch's correction, and bootstrapping were used. No evidence for multicollinearity was found (VIF = 1–2.24).

The results show a small but significant effect of clinical depressiveness on the combination of the decision‐making modes (Model 1: Pillai's trace = 0.009, *F*(6, 4832) = 7.32, *p* < 0.001, η_p_^2^ = 0.009). The effect of clinical depressiveness is still significant after controlling for the covariates. Even though the covariates have strong significant effects, the variance they explain is small (see Table 4).

**TABLE 4:** Results of the MANCOVA (Model 2)

	Pillai's trace	*F*	*df_1_*	*df_2_*	*p*	η_p2_
Clinical depressiveness	0.009	7.45	6	4,821	< 0.001	0.009
Age	0.032	26.72	6	4,821	< 0.001	0.03
Sex	0.098	87.62	6	4,821	< 0.001	0.10
Highest education	0.049	4.94	48	28,956	< 0.001	0.008
Marital status	0.003	2.06	6	4,821	0.054	0.003

NOTE: *N* = 4,839, *n*_CD_ = 278, *n*_CD_ = 4,561.

**FIGURE 1:**
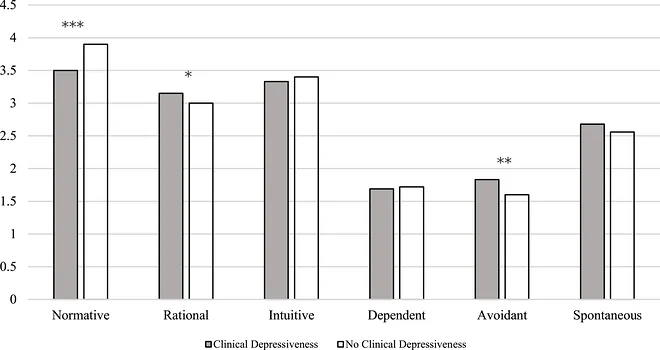
Means of the decision‐making modes by clinical depressiveness

The results of the analysis of variance (ANOVA) show that individuals with clinical depressiveness engage more in the rational and avoidant modes than individuals without clinical depressiveness, whereas those without clinical depressiveness engage more in the normative mode. The results have small‐to‐medium effect sizes (see Figure 1 and Table 5).

**TABLE 5:** Results of the post hoc ANOVA

Decision‐making mode	*F*	*df_1_*	*df_2_*	*p*	Cohen's *d*
Normative	20.27	1	303.76	<0.001	0.31
Rational	4.99	1	311.09	0.026	0.14
Intuitive	0.62	1	309.96	0.431	0.05
Dependent	0.22	1	310.08	0.643	0.03
Avoidant	11.57	1	302.81	0.001	0.24
Spontaneous	2.14	1	307.63	0.145	0.10

Exploratory interaction analyses of the covariates on the relationship between decision‐making modes and clinical depressiveness revealed several significant effects: Individuals without clinical depressiveness showed less agreement with the normative mode as age increased (β = −0.03, *SE* = 0.02, *p* = 0.041). Additionally, those without clinical depressiveness were more likely to perceive having children as a normal part of life if they were married (β = 0.60, *SE* = 0.25, *p* = 0.015) or had an *Abitur* (German high school diploma with university entrance qualification; β = 0.65, *SE* = 0.28, *p* = 0.021). Women compared to men agreed significantly more with the idea that the partner should make the decision (β = −0.72, *SE* = 0.11, *p* < 0.001) and were more likely to postpone the decision (β = −0.36, *SE* = 0.12, *p* = 0.003). Interestingly, women without clinical depressiveness are more likely to postpone the decision than women with clinical depressiveness (β = 0.26, *SE* = 0.13, *p* = 0.041).

### Hypothesis 2

Requirements for the fixed effect regressions and CLPM were partially met: The independent variables showed no multicollinearity (VIF = 1.11), and the relation between independent and dependent variables is linear. There was no autocorrelation, and residuals were normally distributed. There was homoscedasticity in the examined variables (Breusch–Pagan test: *p* > 0.05); Still, robust standard errors (HC3) were used to account for potential violations of this assumption (see the Online Appendix for all results).

**FIGURE 2:**
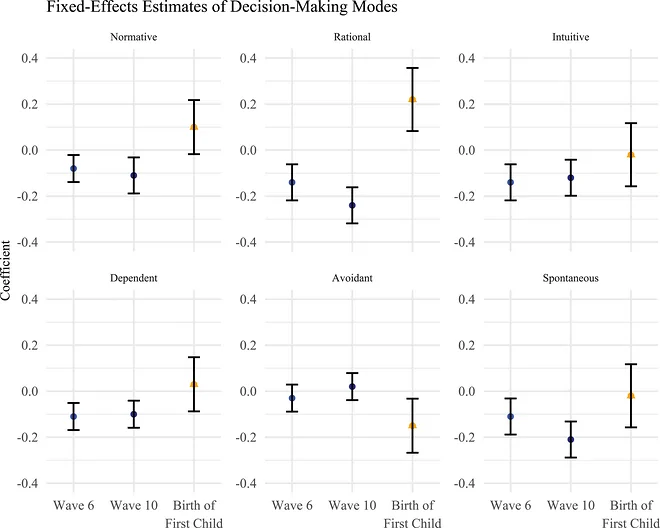
Development of the decision‐making modes with and without the effect of the first child

The results of the fixed effect regression (see Table 6 and Figure 2) show that all decision‐making modes except for the avoidant mode decrease significantly over time when the birth of the first child is not integrated. These fluctuations and the variance explained by these effects are small. The decreases in the decision‐making modes remain even after including the effect of the birth of the first child. Descriptive analyses revealed that 76 percent of participants never had a first child (*n* = 1180) until Wave 10, 12 percent had their first child between Wave 2 and 6 (*n* = 185), and another 12 percent between Wave 7 and 10 (*n* = 187). The birth of the first child had a significant positive effect on the rational mode, thus reducing its negative trend. The birth of the first child also had a significant negative effect on the avoidant mode, indicating changes in the decision‐making mode after the first child was born. The spontaneous mode (6) is the most unstable, whereas the avoidant mode (5) remains stable.

**TABLE 6:** Results of the fixed effect regression (Model 1 + 2)

	Wave 6			Wave 10			Birth of first child			
	β	*SE*	*p*	β	*SE*	*p*	β	*SE*	*p*	*R^2^*
Model 1										
DMM										
1	−0.07	0.03	0.028	−0.09	0.03	0.004				0.29%
2	−0.11	0.04	0.003	−0.18	0.04	0.000				0.70%
3	−0.14	0.04	0.000	−0.12	0.04	0.000				0.58%
4	−0.11	0.03	0.000	−0.09	0.03	0.000				0.58%
5	−0.04	0.03	0.127	−0.02	0.03	0.480				0.08%
6	−0.11	0.04	0.001	−0.21	0.04	0.000				1.15%
Model 2										
DMM										
1	−0.08	0.03	0.011	−0.11	0.04	0.001	0.10	0.06	0.082	0.39%
2	−0.14	0.04	0.000	−0.24	0.04	0.000	0.22	0.07	0.001	1.1%
3	−0.14	0.04	0.000	−0.12	0.04	0.000	−0.02	0.07	0.773	0.59%
4	−0.11	0.03	0.000	−0.10	0.03	0.001	0.03	0.06	0.602	0.59%
5	−0.03	0.03	0.390	0.02	0.03	0.576	−0.15	0.06	0.006	0.32%
6	−0.11	0.04	0.002	−0.21	0.04	0.000	−0.02	0.07	0.825	1.1%

NOTE: DMM = decision‐making mode; *N* = 1552.

The model fit of the CLPM was acceptable while benefiting from improvement. The Root Mean Square Error of Approximation (RMSEA) and Standardized Root Mean Square Residuals (SRMR) had an acceptable fit, and the Comparative Fit Index (CFI) and Tucker Lewis Index (TLI) had values below the established threshold (see the Online Appendix). The results of the CLPM show that the decision‐making modes are stable over time. All autoregressive effects of the decision‐making modes between Wave 2 and 10 were significant at the 0.1 percent level. The normative mode is the most stable, with the highest autoregressive effects. For all other decision‐making modes, the autoregressive effects are moderate. There is a significant positive effect of the birth of the first child on the normative mode, meaning that the birth of the first child increases the attitude that having children is a normal part of life (see Table 7). Other decision‐making modes are not impacted by the birth of the first child. Some decision‐making modes correlate with each other, especially within the same wave (see the Online Appendix), which might indicate dependencies between those modes or influences of confounding variables.

**TABLE 7:** Results of the cross‐lagged panel model

	Waves 2–6		Waves 6–10		First child (Wave 6)		First child (Wave 10)	
DM mode	β	*p*	β	*p*	β	*p*	β	*p*
1	0.54	0.000	0.58	0.000	0.14	0.000	0.15	0.000
2	0.26	0.000	0.30	0.000	−0.03	0.478	0.09	0.053
3	0.28	0.000	0.30	0.000	0.06	0.147	−0.03	0.545
4	0.28	0.000	0.34	0.000	−0.02	0.511	−0.02	0.500
5	0.23	0.000	0.25	0.000	0.01	0.825	−0.07	0.044
6	0.29	0.000	0.33	0.000	−0.05	0.178	−0.07	0.104

NOTE: DM mode = decision‐making mode. *N* = 1552.

Overall, Hypothesis 2 was partially confirmed. The results of the fixed effect regression showed the avoidant mode to be stable within a person. All other modes show significant, but small, within‐person fluctuations. The birth of the first child significantly affected the rational and avoidant decision‐making modes within the person. All other modes were unaffected by the birth of the first child. The fluctuations of the fixed effect regression results disappear when differences between the test subjects are considered. The CLPM indicates relative stability over time; individuals with high scores in a decision‐making mode will have high scores in this mode at a later wave, even though the evaluation fluctuates at the individual level. The CLPM found a significant positive effect of the birth of the first child on the normative mode. Since fixed effect regressions eliminate individual time‐invariant differences, the results suggest an effect of the birth of the first child on the normative mode between test subjects, not within. However, no significant effect of the birth of the first child was found for any other decision‐making mode. Thus, the birth of the first child may not have a large effect on the decision‐making modes between subjects.

Several linear regressions were conducted to exploratory examine possible effects of clinical depressiveness, survey wave, and cohort on the stability of each decision‐making mode (see the Online Appendix). Clinical depressiveness had a significant effect on the avoidant mode only (β = −0.32, *SE* = 0.12, *t* = −2.58, *p* = 0.010), whereas the other decision‐making modes were unaffected by both clinical depressiveness and survey wave. However, participants from cohort 3 and cohort 2 showed effects on different decision‐making modes. Participants from cohort 3 showed significantly lower scores in the normative mode (β = −0.62, *SE* = 0.11, *t* = −5.92, *p* < 0.001) and rational mode (β = −0.20, *SE* = 0.09, *t* = −2.25, *p* = 0.025). They also showed significantly higher scores in the dependent mode (β = 0.18, *SE* = 0.07, *t* = 2.73, *p* = 0.006) and avoidant mode (β = 0.24, *SE* = 0.07, *t* = 3.57, *p* < 0.001). Furthermore, participants from cohort 2 showed significantly lower scores in the dependent decision‐making mode (β = −0.07, *SE* = 0.03, *t* = −1.98, *p* = 0.048) and the spontaneous mode (β = −0.14, *SE* = 0.05, *t* = −3.08, *p* = 0.002) than the reference cohort 1, and significantly higher values in the avoidant decision‐making mode (β = 0.08, *SE* = 0.03, *t* = 2.45, *p* = 0.014).

Additionally, exploratory analyses of gender differences were examined by comparing two CLPM models, one expecting there to be gender differences and one expecting there to be none. A robust chi‐square difference test, comparing both models, showed men and women did not differ in their stability of the decision‐making modes (χ^2^ (12) = 9.20, *p* = 0.686).

## DISCUSSION

The present study shows that individuals with clinical depressiveness engage more in rational and avoidant decision‐making and less in normative decision‐making than individuals without clinical depressiveness. Age, sex, highest education, and marital status showed statistically significant effects, though these explained only a small proportion of the total variance, suggesting limited explanatory power. Decision‐making modes seem relatively stable over time. While individual fluctuations in the decision‐making modes occur, these changes might not impact the overall pattern at an interpersonal level. Distinct decision‐making modes vary with the birth of the first child, depending on inter‐ or intrapersonal observation.

The findings suggest that individuals with clinical depressiveness engage more in avoidant as well as rational decision‐making. This may indicate a capacity to consider reproductive decisions rationally while simultaneously experiencing challenges in the decision‐making process.

First, with respect to the rational decision‐making mode, unique symptomatic aspects of depression may help to understand why individuals with clinical depressiveness engage more in the rational decision‐making mode. People with depression face challenges in the decision‐making process due to dysfunctional decision‐making skills and the difficulty in modifying them into functional ones (Leykin, Roberts, and DeRubeis 2011). Individuals with depression tend to ruminate more, rely less on intuition (Leykin and DeRubeis 2010), engage in negative selective attention (e.g., Bishop and Gagne 2018; Hammar and Ardal 2009; Rock et al. 2014), and adopt negative thought patterns (Freyberger, Dilling, and Weltgesundheitsorganisation 2019). Anhedonia, negative beliefs about oneself, and a negative anticipated decisional outcome have been associated with difficulties in the decision‐making process (Paulus and Yu 2012). Individuals with depressive symptoms engage in an abstract‐analytical rumination mode, which is linked to prolonged decision‐making times and higher levels of indecisiveness (Di Schiena et al. 2013). Thus, even rational decision‐making could be perceived as suboptimal through the lens of depressive symptoms. In the *pairfam* study, rational decision‐making was operationalized through the statement “Personal costs‐and‐benefits driven decision.” Rumination might serve as a strategy to evaluate a decision thoroughly by considering as many possibilities as possible. However, due to the dysfunctional nature of ruminative thoughts, a general negative perspective, and selective attention biases related to depressive symptoms, the rational decision‐making process itself may be experienced as distorted.

Second, the engagement in avoidant decision‐making has been linked to a present low risk tolerance in depression (Leahy 2001). The risk of making a wrong decision might seem lower if no decision is made at all. Individuals with depression anticipate fewer rewarding consequences from their decisions (Bishop and Gagne 2018), and depressive symptoms are significantly associated with negative motives for wanting children (Erbe, Freisinger, and Gerlach 2026). Moreover, they perceive more costs and less benefits in having children than individuals without depression (Erbe and Preetz 2022), potentially making postponement appear as a low‐risk choice. The avoidant decision‐making mode was the only mode that was significantly affected by clinical depressiveness during the exploratory analyses of Hypothesis 2, supporting the idea that avoidant decision‐making may represent a characteristic of individuals with clinical depressiveness. However, the interaction analyses showed contradictory results, indicating women without clinical depressiveness tend to postpone more likely the decision than women with clinical depressiveness. Unequal distributions among men and women in the depressiveness groups could explain these results. In Wave 2, there were 166 women with clinical depressiveness and 2,157 women without clinical depressiveness. Furthermore, additional variables that were not controlled for in the analyses might mediate or moderate these results.

Age, sex, highest education, and marital status showed significant individual effects on the relationship between clinical depressiveness and decision‐making modes. However, they explained only little of the variance, and the association between clinical depressiveness and decision‐making modes remained significant even after controlling for these covariates, suggesting limited overall influence. Nevertheless, the findings indicate that the relationship between depressiveness and reproductive decision‐making, or each variable individually, may be affected by confounding variables. Therefore, the present results should be interpreted carefully. Increased symptoms of depression and anxiety have been associated with individual life circumstances, such as being single, being in a low‐quality relationship (Leach et al. 2013), or experiencing involuntary childlessness (Lechner, Bolman, and Van Dalen 2007). In addition, inconsistencies in contraceptive usage were more frequently observed among individuals with depression compared to those without depression (Kremer, Gerlach, and Erbe 2024). Conversely, factors such as happiness, being in a stable partnership (e.g., marriage), and relationship satisfaction are predictors for higher childbirth intentions and earlier childbirth decisions, with higher age at the beginning of the partnership (Hashemzadeh et al. 2021). If stable relationships predict clearer reproductive intentions (Hashemzadeh et al. 2021), unstable ones may be associated with differences in reproductive decision‐making, perhaps in relation to existing mental health symptoms. Taken together, these findings are consistent with the possibility that additional variables influence depressiveness, reproductive decision‐making, or their interaction, and have to be kept in mind while interpreting the present results.

The present findings suggest an association between clinical depressiveness and reproductive decision‐making. However, it is equally possible that the decision‐making modes associated with clinical depressiveness may also be related to depressive symptoms themselves. For instance, postponing reproductive decisions may be related to a higher likelihood of involuntary childlessness (Schmidt et al. 2012), which has been repeatedly linked to heightened psychological distress, including depressive symptoms (e.g., Lechner, Bolman, and Van Dalen 2007; Ribeiro, Pedro, and Martins 2024). Delayed parenthood or involuntary childlessness may be perceived as a missed developmental milestone (Seiffge‐Krenke 2012), and failing age‐normative developmental tasks has been associated with impaired health outcomes (Havighurst 1953). Therefore, the present findings should be interpreted with caution, particularly in light of the potential for reverse causality.

The decision‐making modes change significantly at an intrapersonal level, and the birth of the first child also had individual, significant effects on distinct decision‐making modes. However, since no fluctuations were found at the interindividual level, their effect appears limited. Pregnancy intentions are often implicit and unclear (Helfferich et al. 2021). However, starting a family nowadays involves various considerations, such as personal, financial, or temporal resources (Rupp 2005; Rupp and Bierschock 2005). These considerations may be related to decision‐making at a personal level. The onset of clinical symptoms, changes in the partnership, financial situation, or the birth of another child may contribute to fluctuations at an individual level. Since the individual cohort of participants was related to distinct decision‐making modes, reproductive decision‐making appears to vary with individual historical and generational socialization. Social norms, roles, and needs of the individual generation may be associated with reproductive decisions.

### Strengths and Limitations

First, the *pairfam* study offers an extensive dataset, several survey waves, and a large, representative sample, which strengthens the reliability and representativeness of the present results. The longitudinal analyses and large sample used in the present study, even after excluding missing data, further enhance the quality of the results. However, large sample sizes are also associated with small effects becoming statistically significant.

Second, depressiveness was assessed via a well‐validated and reliable questionnaire, which allowed the operationalization of clinical depressiveness. Besides its breadth, the *pairfam* dataset sometimes suffers due to shortened questionnaires and survey questions. Depressiveness was the only clinical pattern measured using a valid questionnaire. However, the STDS‐t is only a screening questionnaire. Thus, it does not allow a categorical diagnosis of depression. A subsequent integration of well‐established clinical questionnaires could provide more nuanced insights.

Third, the present study offers valuable fundamental insights into the decision‐making process during family planning. In the research context, this is particularly relevant to developing accurate aspects and needs of support programs. Thereby, the present study offers a theoretical foundation to improve guidance in the clinical context. A broader evaluation of the decision‐making processes on reproductive behavior could be a subject of future research. Since the examined decision‐making modes established in *pairfam* might not display the full decision‐making process, a thorough exploration of those would be useful.

Fourth, longitudinal data allow for the examination of the stability of the decision‐making modes over time, thereby offering insights into their development before and after the birth of the first child. However, other aspects of life, such as the onset of clinical symptoms or the decision for another child, may be worth investigating as well. Furthermore, until now, only three survey waves assessed the decision‐making modes. It would be interesting to explore their stability over a longer time period.

Fifth, the present findings suggest an impact of clinical depressiveness on reproductive decision‐making processes that has not yet been examined in this way. However, it is equally possible that decision‐making modes such as postponing the decision may simultaneously influence depressive symptoms, highlighting the need for careful interpretation of the findings. Potential confounding variables such as age or relationship status may moderate or mediate the relationship between depressiveness and reproductive decision‐making, or independently influence the examined variables. Therefore, the direction of the association is unclear. A bidirectional relationship, or the influence of unmeasured third variables, cannot be ruled out. Future research should focus on examining potential confounding variables and the possibility of reverse causality between depressiveness and reproductive decision‐making.

## CONCLUSION

People with clinical depressiveness differ in regard to their engagement in decision‐making processes when it comes to family planning, compared to those without clinical depressiveness. Their decisions are more often characterized by cost–benefit considerations or put off entirely. In contrast, people without clinical depressiveness claim more often that having children is a normal part of life. These decision‐making patterns seem to be stable, even after the birth of the first child, but may fluctuate at an individual level. It is possible that depression‐related characteristics, such as extensive rumination and low risk‐tolerance, may co‐occur with a stronger tendency towards rational and avoidant decision‐making. Furthermore, individual life and generational aspects, as well as changes in circumstances, may be related to the stability of the decision‐making processes. Simultaneously, the findings should be interpreted cautiously with regard to causality, as certain reproductive decisions or individual life circumstances may be associated with changes in depressive symptoms as well. Thus, future research might benefit from incorporating additional social and sociodemographic aspects into their analyses, as well as exploring other clinical conditions to identify further distinctions in decision‐making. Future research could examine whether a family planning guidance targeting individual decision‐making processes is linked to higher long‐term satisfaction with reproductive decisions. Additionally, a qualitative exploration of decision‐making processes and characteristics could provide valuable insights. Lastly, research on the long‐term effects of the decision‐making processes and the examination of longer longitudinal episodes might be interesting.

Overall, the present study introduces valuable, fundamental information by offering deeper insights into a complex and existential topic. In doing so, it paves the way for developing relevant guidance throughout the family planning process and establishes a meaningful and relevant foundation for future research.

## CONFLICT OF INTEREST STATEMENT

All authors declare that they have no competing interests.

## ETHICS APPROVAL STATEMENT AND PATIENT CONSENT STATEMENT

The present study used data from *pairfam* only. *Pairfam* was approved by the ethics committee of the Faculty of Management, Economics and Social Sciences of the University of Cologne. All participants of the *pairfam* study consented to their participation in the *pairfam* study. The authors declare that all procedures contributing to this work comply with the ethical standards of the relevant national and institutional committees on human experimentation and with the *Declaration* of *Helsinki of* 1964, as revised in 2024. In addition, all analyses were conducted under the terms and privacy regulations of *pairfam*.

## Supporting information




Supporting Information

## Data Availability

The dataset analyzed in the present study is partially publicly available (https://www.pairfam.de/en/data/data‐access/). Full access requires a data use agreement and must be requested directly from the *pairfam* research team. R Scripts of statistical analyses can be requested from the corresponding author.
