# Supplementary material for: Decision‐Making and Family Planning among People with Clinical Depressiveness—Findings from a Representative German Panel Study
Source: Stud Fam Plann. 2026 Jul 15;57(3):415–31. doi: 10.1111/sifp.70060 (PMC13578845; doi:10.1111/sifp.70060)
Supplement: Supplementary file 1 [file SIFP-57-415-s001.docx]

**Supplemental Materials**

**Requirements of MANCOVA**

T1	Multicollinearity via Correlations Matrix (*r*)

|  | Mode 1 | Mode 2 | Mode 3 | Mode 4 | Mode 5 | Mode 6 |
| --- | --- | --- | --- | --- | --- | --- |
| Mode 1 | 1 | -0.05 | 0.09 | 0.05 | -0.08 | -0.01 |
| Mode 2 | -0.05 | 1 | 0.08 | 0.05 | 0.10 | 0.01 |
| Mode 3 | 0.09 | 0.08 | 1 | 0.11 | 0.08 | 0.11 |
| Mode 4 | 0.05 | 0.05 | 0.11 | 1 | 0.24 | 0.12 |
| Mode 5 | -0.08 | 0.10 | 0.08 | 0.24 | 1 | 0.13 |
| Mode 6 | -0.01 | 0.01 | 0.11 | 0.12 | 0.13 | 1 |

T2	Multivariate Normal Distribution

|  | estimate | *p* |
| --- | --- | --- |
| Henze-Zirkler Test | 10.98 | .000 |

T3	Homogeneity of Covariance Matrices

|  | *Chi^2^* | *df* | *p* |
| --- | --- | --- | --- |
| Box M Test | 58.59 | 21 | .000 |

T4	Homogeneity of Variance: Levene Test

| Mode | *F* | *df1* | *df2* | *p* |
| --- | --- | --- | --- | --- |
| 1 | 17.4 | 1 | 4837 | .000 |
| 2 | 5e-04 | 1 | 4837 | .981 |
| 3 | 0.078 | 1 | 4837 | .779 |
| 4 | 0.22 | 1 | 4837 | .636 |
| 5 | 14.94 | 1 | 4837 | .000 |
| 6 | 0.26 | 1 | 4837 | .610 |

T5	Multicollinearity: VIF Test

| Variable | VIF value |
| --- | --- |
| Clinical depressiveness | 1.01 |
| Age | 2.24 |
| Sex | 1.03 |
| Highest education | 2.11 |
| Marital status | 1.15 |

**Requirements Fixed Effect Regression and CLPM**

T6	Breusch Pagan Test

| Mode | estimate | *df* | *p* |
| --- | --- | --- | --- |
| 1 | 0.70 | 2 | .705 |
| 2 | 2.63 | 2 | .268 |
| 3 | 3.94 | 2 | .140 |
| 4 | 0.30 | 2 | .863 |
| 5 | 0.54 | 2 | .765 |
| 6 | 0.33 | 2 | .850 |

T7	Durbin Watson Test

| Mode | estimate | *p* |
| --- | --- | --- |
| 1 | 2.03 | .826 |
| 2 | 2.02 | .797 |
| 3 | 2.03 | .806 |
| 4 | 2.03 | .840 |
| 5 | 2.03 | .828 |
| 6 | 2.03 | .814 |

T8	Multicollinearity: VIF Test

| Variable | VIF value |
| --- | --- |
| Birth of first child | 1.11 |
| Wave | 1.11 |

T9	Model Fit CLPM

|  | estimate | *p* |
| --- | --- | --- |
| χ^2^(122) | 781.95 | <.001 |
| CFI | .815 |  |
| TLI | .741 |  |
| SRMR | .056 |  |
| RMSEA  90% CI – lower  90% CI – upper | .059  .055 | <.001  <.001 |

*Note. CFI, TLI, RMSEA: robust values used.*

**Exploratory Analyses**

T10	Correlation between the Decision-Making Modes and Waves

|  | *Wave 2* | | *Wave 6* | | *Wave 10* | |
| --- | --- | --- | --- | --- | --- | --- |
| Mode 1 x | *r* | *p* | *r* | *p* | *r* | *p* |
| Mode 2 | -0.102 | 0.008 | -0.034 | 0.252 | -0.100 | 0.004 |
| Mode 3 | 0.141 | <.001 | 0.080 | 0.010 | 0.131 | <.001 |
| Mode 4 | 0.045 | 0.096 | -0.019 | 0.385 | -0.029 | 0.206 |
| Mode 5 | -0.130 | <.001 | -0.104 | <.001 | -0.089 | <.001 |
| Mode 6 | -0.061 | 0.136 | -0.007 | 0.830 | -0.011 | 0.726 |

*Note. Mode = Decision-Making Mode.*

T11	Exploratory Linear Regression: Influence of Clinical Depressiveness, Wave, Cohort

|  | Mode | | | | | |
| --- | --- | --- | --- | --- | --- | --- |
|  | 1 | 2 | 3 | 4 | 5 | 6 |
| CD  β  *SE*  *p* | 0.06  0.15  .672 | -0.27  0.16  .095 | -0.12  0.16  .455 | -0.01  0.12  .952 | -0.32  0.12  .010 | -0.17  0.16  .287 |
| Wave  β  *SE*  *P* | -0.04  0.03  .184 | -0.06  0.04  .160 | 0.03  0.04  .475 | 0.01  0.03  .693 | -0.01  0.03  .829 | -0.00  0.04  .969 |
| CD x Wave  β  *SE*  *p* | 0.02  0.02  .314 | 0.02  0.02  .389 | -0.02  0.02  .274 | -0.01  0.02  .439 | 0.00  0.02  .890 | -0.01  0.02  .535 |
| Cohort 2  β  *SE*  *p* | -0.03  0.05  .518 | -0.08  0.05  .074 | -0.04  0.05  .430 | -0.07  0.03  .048 | 0.08  0.03  .014 | -0.14  0.05  .002 |
| Cohort 3  β  *SE*  *p* | -0.62  0.11  .000 | -0.20  0.09  .025 | -0.13  0.09  .170 | 0.18  0.07  .006 | 0.24  0.07  .000 | -0.01  0.09  .875 |

*Note.* Mode = Decision-Making Mode, CD = Clinical Depressiveness
